# CENet: A Cabinet Environmental Sensing Network

**DOI:** 10.3390/s100201021

**Published:** 2010-01-28

**Authors:** Zusheng Zhang, Fengqi Yu, Liang Chen, Guangmin Cao

**Affiliations:** 1 Institute of Computing Technology, Chinese Academy of Sciences, No.6 Kexueyuan South Road Zhongguancun, Haidian District Beijing, China; 2 Shenzhen Institute of Advanced Technology, Chinese Academy of Sciences, 1068 Xueyuan Avenue, Shenzhen University Town, Nanshan District, Shenzhen, China; E-Mails: fq.yu@siat.ac.cn (F.Y.); liang.chen@siat.ac.cn (L.C.); gm.cao@siat.ac.cn (G.C.); 3 Graduate University of Chinese Academy of Sciences, No.9 Yuquan Road, Sijingshan District Beijing, China

**Keywords:** wireless sensor network, cabinet monitoring, data-aided routing protocol, scheduling

## Abstract

For data center cooling and intelligent substation systems, real time cabinet environmental monitoring is a strong requirement. Monitoring data, such as temperature, humidity, and noise, is important for operators to manage the facilities in cabinets. We here propose a sensing network, called CENet, which is energy efficient and reliable for cabinet environmental monitoring. CENet achieves above 93% reliable data yield and sends fewer beacons compared to periodic beaconing. It does so through a data-aided routing protocol. In addition, based on B-MAC, we propose a scheduling scheme to increase the lifetime of the network by reducing unnecessary message snooping and channel listening, thus it is more energy efficient than B-MAC. The performance of CENet is evaluated by simulations and experiments.

## 1. Introduction

Cabinet environmental monitoring represents a typical application for wireless sensor networks [1,2]. From data center to substation, there are many cabinets and dedicated containers. These cabinets usually contain computers, high voltage switches, or other electronic equipments. Cabinet environmental monitoring is a technology that provides real-time recording of environment parameters in the interior of cabinets, such as temperature, humidity, smoking, noise, and so on. Depending on these data, operators can estimate the health and running status of the equipment in the cabinets. For example, a thermal alarm means a danger of potential hot spots. Operators need to identify the problem and make intelligent decisions on facility management.

A data center or substation can have several rooms. Each room can have dozens of cabinets. And every cabinet may have several racks. Operators need real-time monitoring of the equipment on each rack. In a substation, there are hundreds of cabinets which contain many kinds of electronic equipment, therefore huge numbers of points need to be monitored. Traditional solutions for cabinet environmental monitoring involve the use of wired sensors, connected through Ethernet interfaces [3]. However, those approaches suffer from high installation and configuration costs. Furthermore, sensor deployment is constrained by the availability of Ethernet connections.

Wireless sensor network (WSN) [4] is an ideal candidate for cabinet environmental monitoring because it is low cost and unattended technology. It can provide wide and dense coverage, and can be easily reprogrammed. Wireless sensors do not require network facility infrastructure in data centers or substations which is already a complicated environment with large amounts of equipment.

Cabinet monitoring introduces new challenges to wireless sensor networks. The network framework must have good scalability to support new equipment. Commands have to be reliably disseminated throughout the entire system so that all parts of the network can follow the commands to change their states. Sensed data must be reliably collected, e.g. temperature alarm information is too valuable to be lost due to communication errors or collisions. For battery powered wireless sensor networks, the network lifetime is a critical factor because replacing batteries for sensor nodes is a laborious task.

In this paper, we present CENet (Cabinet Environmental sensing Network). Focusing on network lifetime and reliability of data transmission, CENet provides two main contributions:

- CENet implements a data-aided routing protocol. Instead of using periodic beaconing to estimate link quality and maintain network routing topology, CENet uses transmitted data to continuously estimate link quality and its routing maintenance is triggered when poor link quality is detected. Therefore the energy dissipation due to beacons can be diminished. Compared to MintRoute’s fixed 1 minute beacon interval [5], CENet sends 85% fewer beacons. It can also achieve a packets delivery ratio of more than 93%.
- CENet contains two kinds of nodes: master nodes and slave nodes. In order to prolong the network life time, we propose a scheduling scheme based on B-MAC [6] for slave nodes, which reduces unnecessary message snooping and channel listening, *i.e.*, a slave node keeps sleeping until it is time for sensing the environment or it has data for transmission. Using CENet, slave nodes consume about 65% less energy compared to using B-MAC, where the sample interval is 5 minutes.

The remainder of this paper is organized as follows. Section 2 gives the system architecture of cabinet environmental monitoring. Section 3 presents the design of routing protocol. Section 4 describes the scheduling scheme. Section 5 evaluates the performance of CENet by simulations and experiments. Section 6 describes related works. Finally, Section 7 gives a brief conclusion.

## 2. Architecture

**Figure 1.**
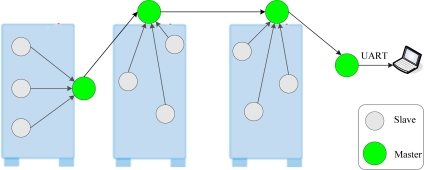
CENet topology.

**Figure 2.**
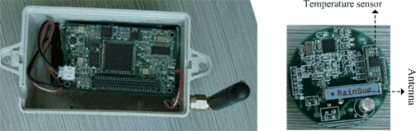
Mica2 node and Dot node.

CENet uses a hierarchical architecture, as shown in Figure 1. The gray circles denote slave nodes and the green circles denote master nodes. It has one base station (BS) which connects a computer through UART. We use Mica2 node as master and Dot node as slave, as shown in Figure 2. The node parameters are shown in Table 1. Slave nodes are placed inside cabinets and master nodes are typically installed outside of cabinets. Slave nodes are responsible for sensing data and transmitting packets to their master nodes. Each slave node can select only a master node as its parent. Master nodes form a routing tree and are responsible for forwarding data to the BS. They are not responsible for sensing the environment. In other words, slave nodes are the sensing front tier and master nodes are the communication infrastructure. The communication distances of slave nodes and master nodes are 0–3 meters and 0–15 meters, respectively. This short distance has the advantage of reducing communication interference because nodes are densely deployed.

The communication stack of CENet is as follows. A modified B-MAC [6] is used as MAC protocol. Mode 4 of B-MAC is used for master node scheduling, while our proposed scheduling based on B-MAC is used for slave node scheduling. The proposed data-aided routing protocol is used for both types of nodes. And the application layer contains two operations: data collection and event alarm.

## 3. Routing

**Figure 3.**
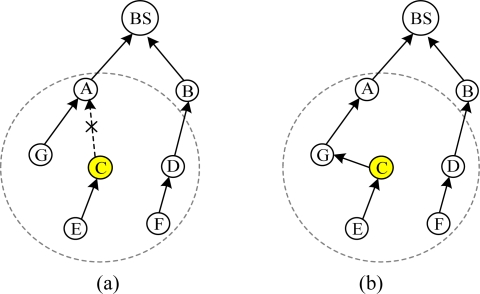
(a) The link from C to A is failed. (b) C selects G as its parent.

**Figure 4.**
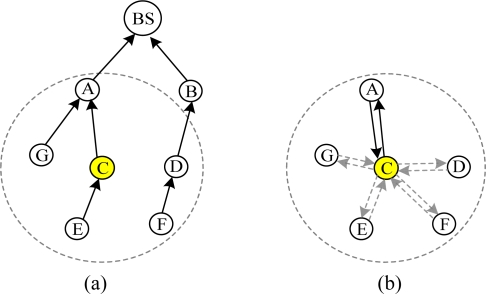
(a) A routing topology. (b) Focus on the nodes in the circle of (a). For node C, only the bidirectional link between C and A effects the reliability of packet transmission.

Most existing routing protocols such as MintRoute [5], MultihopLQI [7], Directed Routing [8], Backbone Routing [9], etc. use periodic beaconing to estimate the link quality and maintain routing topology. Each node periodically measures the link qualities between itself and its neighbors by calculating the expected transmission (ETX [10]). The periodic beaconing has the following drawbacks:

1. Periodic beaconing has a slow response to network variation. The link quality is a statistical result, so it must to be estimated after many beacon exchanges for more accuracy. In some situations, these beacon exchanges should be finished in a short time. For example, when a node parent has failed, the node needs to find a new parent right away. However, taking into account the energy efficiency, the protocols using periodic beaconing should not use short intervals. As shown in Figure 3, when the link from C to A is failed, C needs to select a new parent. Assume at least eight rounds of beaconing are needed to accurately estimate link quality. The default beaconing interval in MintRoute is 8 s and at least 64 s are needed to recover the failure route. CENet solves the problem by considering a network change as an event. When an event is detected, route maintenance is triggered. Many beacons are exchanged in a short time and failed routes can be quickly repaired.
2. Periodic beaconing is not energy efficient. A node periodically broadcasts beacons to its neighbors without considering link quality and data transmission. We denote the link from a node to its parent as L2P. A node selects the neighbor node with the best link as its parent. When L2P quality is good enough to support data transmission reliability, it is not necessary to change parent. As shown in Figure 4 (a), the parent of node C is A and only the bidirectional link between C and A effects their communication reliability. Let’s focus on the nodes inside the circle, as shown in Figure 4 (b). Node C broadcasts beacons to its neighbors and estimates the link qualities periodically. But for the link between C and A, the beacon exchanges between node G, D, E and F are redundant and waste energy. CENet solves the problem as follows: when a node is trying to find a parent, the link qualities of all the routes connected to the BS are estimated using beacons. When the node has found its parent, it stops issuing beacons. The link quality variation of L2P is monitored by transmitted data.

Our data-aided routing protocol can overcome the shortcomings of the current periodic beaconing routing protocols. It is more energy efficient and reliable for data packet transmission.

### 3.1. Routing Tree Construction

In CENet, beacons are divided into three categories: request, reply and pull. All of these beacons are transmitted by broadcasting. A request beacon is to advertise that the sender doesn’t have a proper parent and it is searching for a parent. A reply beacon is to indicate that the sender has a route to BS and can be a candidate parent. Finally, a pull beacon indicates that the sender has better or updated route information and notifies its neighbors to maintain their routes.

When a node does not have a parent or its parent turns to fail, it starts searching for a parent by periodically broadcasting request beacons to its neighbors. A neighbor node with a determined parent broadcasts a reply beacon when it receives the request. The request and reply beacons need to exchange many times to accurately estimate the link qualities. We use expected transmission (ETX [10]) as a cost metric, which reflects the quality of the link to one neighbor. The more reliable the link quality is, the smaller the ETX. The route cost is the sum of link ETX values along the entire route. A node selects a parent with the least route cost. Once a node has found its parent, it stops sending requests.

The frequency of sending request beacons is determined by user requirements. For cabinet monitoring application, if a user wants to construct a routing tree quickly after sensor deployment, they can set the interval to several hundreds of milliseconds.

After sensor deployment, only BS has a parent which is an upper computer connected over UART. Other nodes do not have parents and broadcast requests periodically. When BS receives the requests from the nodes closer to it, it sends reply beacons. After enough exchanges of the beacons, the sensor nodes at the 1^st^ level in the routing tree choose BS as parent and stop sending requests. Similarly the nodes at the 2^nd^ level exchange request and reply beacons with the nodes at the 1^st^ level to determine their parent. The whole routing tree can be constructed after several rounds. When the routing tree is constructed, none of the nodes in the network sends request beacons. If the communication links in the network remain good, there will be no beacon exchange at all and the link quality is estimated by transmitted data.

### 3.2. Link Quality Estimation

Since CENet uses acknowledge and retransmission schemes, the decline of link quality increases the number of retransmissions and packet drops. The link quality is estimated by observing the number of packet retransmissions. CENet uses the window mean with EWMA (exponentially weighted moving average) as link quality estimator [11] which computes an average retransmission rate over a time period and smoothes the average by EWMA.

Let *i* be the index of a time window, and *t* be the time window represented in the number of message transmissions and *C_i_* be the number of packet retransmissions in the *i^th^* time window. *H_i_* is a value between *C_i_* and history retransmissions, *H_i_* can be expressed as:

(1) $$H_{i}=\{\begin{array}{cc} 0, & i=0\\ C_{i,} & i>0\;\mathrm{and}\;H_{i-1}=0\\ H_{i-1}*a+\left(1-a\right)*C_{i}, & i>0\;\mathrm{and}\;H_{i-1}>0 \end{array}$$

(2) $$T_{i}=\left\lceil H_{i}*\left(1+b\right)\right\rceil$$

where 0 < *a* < 1 and 0 < *b* < 1 both are tuning parameters, *T_i_* is a threshold. In the time window *t*, if *H_i_* is greater than *T_i-1_*, the route maintenance is triggered and *H_i_* will be reset to 0 in next round estimation. To understand (1) and (2), we give an example, let *C_i_* be a random value between 7 and 10, *a* = 0.5 and *b* = 0.1. Table 2 shows the retransmission result of five time windows. In the 3^rd^ and 5^th^ windows, the number of retransmissions is greater than the threshold and routing maintenance is triggered.

From Table 2, we can see a problem in the link estimator. When *T_i-_*_1_ = 0, *C_i_* has a useless reference value and the estimator will never be able to detect the decrease of link quality in the current time window. To solve the problem, we set two criteria for routing maintenance. One is the estimator mentioned above. The other is data packet drop rate. For example, when the packet drop rate is greater than 10% (according to the reliability level of the user requirement) in a time window, the routing maintenance process will be triggered.

### 3.3. Routing Tree Maintenance

In our data-aided routing protocol, the route maintenance of a node is triggered under the following events:

- When a node’s link quality to its parent gets worse or the link has failed.
- When a node receives a pull beacon.

After routing maintenance has been triggered, a node sends request beacons periodically to select a proper parent. If it finds a parent and the route cost to BS is significantly decreased (we choose 20% decrease as threshold), the node broadcasts this event using pull beacons because it may provide a better route for nearby nodes. On the other hand, if the new route cost is significantly increased (we choose 20% increase as threshold) or it cannot find a parent, the node also informs the event to its children using pull beacons so that they will adjust the network topology in time.

**Figure 5.**
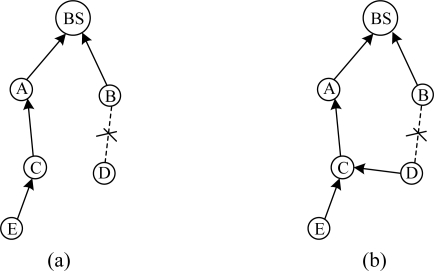
Declining of link quality triggers routing maintenance.

The scene of route failure is demonstrated in Figure 5 (a). The link quality between node D and its parent B is estimated to be worse by transmitted data packets, and routing maintenance is triggered. As shown in Figure 5 (b), C is selected as its new parent.

**Figure 6.**
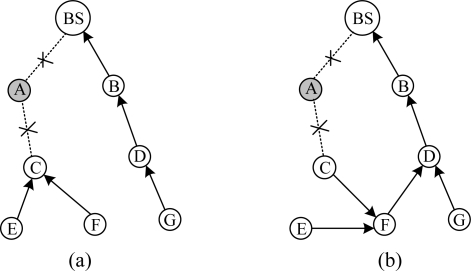
Parent node informs its children to trigger routing maintenance.

As shown in Figure 6 (a), when node C detects that node A is removed or failed, it can’t find an available parent in its communication range. C tells its children E and F that the route is invalid by broadcasting pull beacons. Routing maintenance of E and F is triggered and a new routing tree is reconstructed as shown in Figure 6 (b).

**Figure 7.**
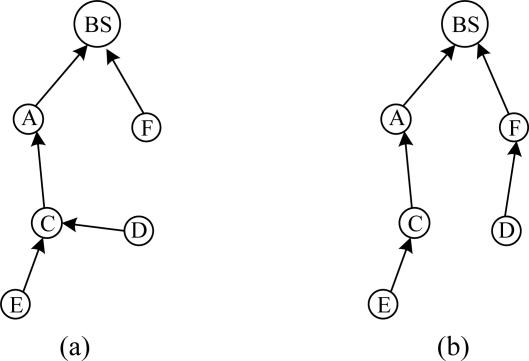
When a new node F joins network, routing maintenance is triggered.

When a new node joins network, routing maintenance is triggered. As shown in Figure 7 (a), node F is a new node and its parent is BS. F broadcasts pull beacons. D receives the pull beacons and routing maintenance process is triggered. Eventually D chooses F as its new parent, as shown in Figure 7 (b). If the change in link cost is significant (20%), D will inform its neighbors the cost change by pull beacons.

## 4. Scheduling

Radio communication dominates the power consumption in wireless sensor networks [12]. Sensor nodes waste a lot of energy for receiving signals. The energy consumption for receiving is approximately equal to that for data transmission. In addition, a node in idle mode also consumes energy. Therefore, idle listening, the time spent for waiting packets, consumes a significant amount of energy. Stemm *et al.* [13] observed that idle listening dominated the energy consumption of network interfaces in hand-held devices. It becomes clear that using scheduling to reduce listening and turn off the radio during idle time is an effective way to prolong network lifetime.

### 4.1. Modified Scheduling Scheme Based on B-MAC

There are two kinds of scheduling: (1) Integrated scheduling with MAC layer, such as B-MAC [6], S-MAC [14]; (2) Independent scheduling based on routing topology, such as FPS [15], DB-TDMA [16], and DCS [17]. Independent scheduling protocols need time synchronization and energy consuming. CENet adopts B-MAC which has friendly interfaces that allow users to flexibly control the operation of scheduling. It has better performance than S-MAC.

For hierarchical architecture of cabinet monitoring, a master node can’t forecast when to receive data packets from its children. In B-MAC, to ensure sensed data being forwarded to the BS reliably, master nodes must detect data when each of its neighbors transmits a packet, regardless of the packet destination. In CENet, mode 4 of B-MAC is selected as the scheduling of master node, which is the optimum mode of B-MAC. Master nodes wake up every 100 ms to detect channel activity. On the other hand, slave nodes, which do not forward data, can decide when to send data. Based on B-MAC, we have developed a scheduling scheme for slave nodes.

**Figure 8.**
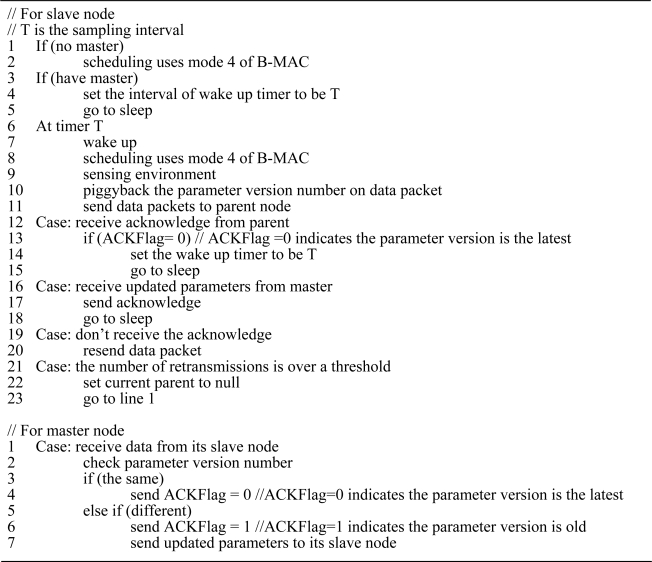
The pseudo code of the proposed scheduling.

The proposed scheduling must support beacon and command transmission. Figure 8 describes the pseudo code of the scheduling. Each node has two states: sleep and active. When a slave node needs to communicate with its master, it enters active state. Because communication nodes must be in the same mode in B-MAC, the scheduling for active state is mode 4 of B-MAC. A slave node wakes up at predetermined time to sense the environment and sends data to its master. Meanwhile, it piggybacks the parameter version number on data packet to check whether the parameters are the latest. If its master has a new version of parameters, it sends updated parameters to its slave immediately. If the number of data packet retransmissions or drop rate is over a threshold, the slave node starts to look for a new master. Otherwise the slave node goes to sleep.

### 4.2. Analysis of Energy Consumption

In this section, we compare the energy consumption between mode 4 of B-MAC and our modified scheduling scheme. Ignoring the energy consumption in parameter update and beacon exchange, the energy consumption of a slave node consists of energy consumed by transmission (*E_tx_*), data sensing (*E_s_*), receiving (*E_rx_*), listening (*E_listen_*), and sleep (*E_sleep_*). The related parameters [6] are shown in Tables 3 and 4, where the average listening current and listening time are obtained in our experiment which will be discussed in detail in the second paragraph of Section 5.2.2. Taking the sample interval as 5 minutes, we can calculate the energy consumption of a slave node in a sample interval, denoted as *E*

(3) $$E=E_{tx}+E_{s}+nE_{rx}+E_{\mathrm{listen}}+E_{\mathrm{sleep}}$$

(4) $$E_{\mathrm{listen}}=I_{\mathrm{listen}}T_{\mathrm{listen}}V$$

(5) $$T_{\mathrm{listen}}=t_{\mathrm{listen}}N_{\mathrm{listen}}$$

(6) $$E_{\mathrm{sleep}}=I_{\mathrm{sleep}}T_{\mathrm{sleep}}V$$

(7) $$T_{\mathrm{sleep}}=T-t_{x}-t_{s}-nt_{rx}-T_{\mathrm{listen}}$$

where *n* is the number of neighbors; *T* is the sample interval and it is equal to 5 min × 60 = 300 s; *N_listen_* is the number of listening times in *T*; *T_listen_* is the total listening time in *T*; *T_sleep_* is the total sleep time in *T*.

Using mode 4 of B-MAC, the default value of channel listening interval is 0.1 s, and the period of a listening is *t_listen_* = 0.01 s. In five minutes, *N_listen_* = 5 min × 60/ (0.1 s + 0.01 s) = 2,727 and *T_listen_* = 27.27 s, so *E_listen_* = 163.6 *mJ*. In B-MAC, a node can detect and receive data packet transmitted from its neighbors. If each of its neighbors sends a data packet, the node will receive *n* packets. Given *n* = 3, the energy consumption for receiving the *n* packets is 17.24 *mJ. T_sleep_* can be calculated as *T_sleep_* = 271.12 *s* and *E_sleep_* = 24.40 *mJ*, so *E* = 278.92 *mJ*. Using mode 4 of B-MAC, the energy consumption for listening and data receiving takes 58.6% and 6.2% of the total energy dissipation, respectively.

While using the modified scheduling scheme, a slave node does not need to receive data packets, neither listening to the channel. The energy consumption of a slave node only consists of the energy consumption for data sampling (*E_s_*), data transmission (*E_tx_*), and sleep (*E_sleep_*). We can calculate *T_sleep_* = 298.873 s, *E_sleep_* = 26.898 *mJ*, and *E* = 100.5613 *mJ*. Therefore CENet consumes about 65% less energy compared to mode 4 of B-MAC when the sample interval is 5 minutes.

## 5. Evaluation

### 5.1. Simulations

In this section, CENet is evaluated in simulations. A prototype was implemented in TinyOS [18]. CENet system is compared to standard TinyOS networking stack (MintRoute [5] on top of B-MAC [6]), standard stack for short, in terms of reliability and energy efficiency. TOSSIM [19] is used in all simulations, which is a discrete event-based simulator for TinyOS. TOSSIM requires a user specify the signal attenuation levels for every network link. We used the Log Distance Path Loss model to calculate these attenuations [20]. One hundred and one nodes are deployed in an area of (50 m, 50 m). There were 70 slave nodes, 30 master nodes, and one BS placed at (0, 0).

**Figure 9.**
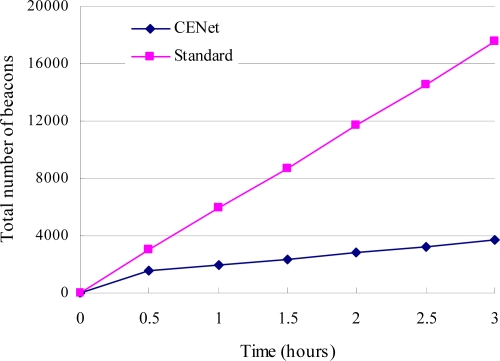
The number of beacons for CENet decreases after a period of time. It is significantly less than that of standard stack.

The number of beacons for CENet and standard stack are shown in Figure 9. The beaconing rate of CENet is high during network startup because every node is busy with exchanging beacons to discover the routes and construct the network. The beaconing rate decreases after a period of time, because in CENet beacons are only sent when the route needs be maintained. In MintRoute, beacons are sent at a fixed interval of 1 minute, which is a standard stack routing protocol. CENet has much less number of control packet transmissions than MintRoute.

**Figure 10.**
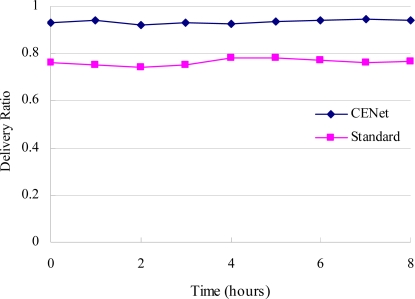
CENet has a consistently higher delivery ratio than standard stack.

Figure 10 shows the simulation results when CENet and standard stack are run for over 8 hours, respectively. The maximum number of retransmissions for CENet and standard stack are both 4. The delivery ratio for CENet and standard stack are 93% and 76%, respectively. Therefore CENet has higher reliability than the standard stack.

**Figure 11.**
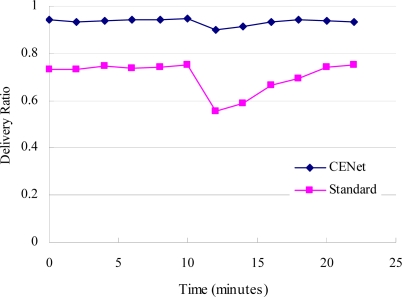
Robustness of CENet and standard stack when five master nodes are removed in the 10th minute.

The ability of route failure recovery is studied by running CENet and the standard stack for 25 minutes with a packet transmission interval of 10 s. After 10 minutes, we remove five master nodes that are forwarding most of the packets in the network. The simulation results are shown in Figure 11. It shows that CENet has only a small drop in delivery ratio at 12 min. This is because when a node detects a route failure, it transmits beacons periodically with an interval of several hundreds milliseconds. Thus the failure route can be repaired quickly. However, the delivery ratio for standard stack is 55%. This is because its routing protocol is MintRoute that sends beacons every 1 minute. Therefore it needs several minutes to repair the failure.

**Figure 12.**
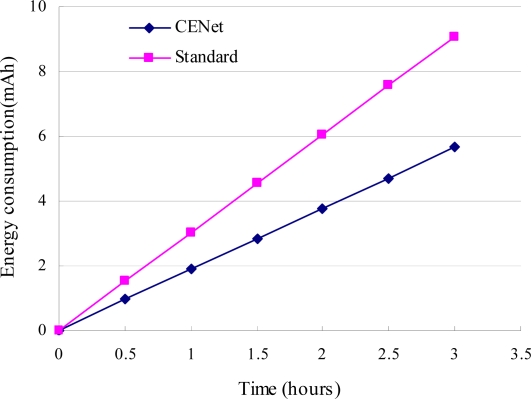
Energy consumption of CENet and standard stack.

**Figure 13.**
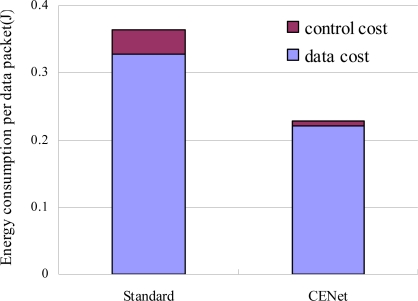
The energy consumption of CENet is 38% less than that of standard stack and the energy consumption for control packets in CENet is 85% less than that in standard stack.

The energy consumption for CENet and standard stack is studied by the following simulations. Slave nodes generate sensing data every 30 seconds and MintRoute beaconing interval is 1 minute. Figure 12 shows that the average node energy consumption is 9.1 mAh for the standard stack and 5.65 mAh for CENet over 3 hours. Therefore CENet is about 38% more energy efficient than the standard stack. Figure 13 compares the energy consumption of delivering each data packet from source node to BS for CENet and standard stack. It shows that control packets for CENet are much less than those for the standard stack (3% *vs.* 7.2%). The decrease in the number of control packets is due to the data-aided routing protocol, where beacon exchange happens only when route needs to repair.

**Figure 14.**
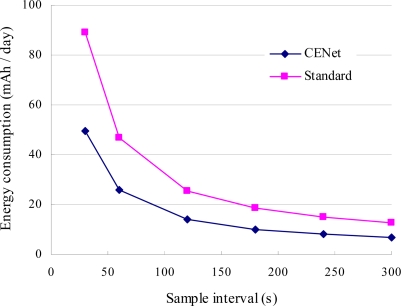
Average energy consumption of CENet and standard stack for sample interval between 30 s and 300 s.

CENet and standard stack are evaluated in terms of overall network lifetime. Considering the tradeoff between energy consumption and response time to network change, let the beacon interval of MintRoute be equal to the sample interval of slave nodes, which varies from 30 s to 300 s. As shown in Figure 14, when the sample interval is 30 s, the average energy consumption of slave node is 49 mAh/day for CENet and 86 mAh/day for standard stack. So a pair of AA batteries (2,500 mAh) can serve 51 days for a node running CENet and 29 days for running the standard stack. When the sample interval is 5 minutes, the average energy consumption of each node is 6.9 mAh/day for CENet and 12.8 mAh/day for standard stack. So the network lifetime can be longer than one year for a CENet network.

### 5.2 Experiments

#### 5.2.1. Cabinet Environmental Deployment

CENet monitors the temperature of the isolated contacts in high voltage cabinets. The cabinet is steel and has a plastic window for the operator to see the equipment running state, so the RF signal can transmit through the window. Slave nodes are deployed in the cabinets for temperature sensing. Master nodes are placed outside the cabinet.

Thirty six nodes are deployed in high voltage cabinets in a substation. There are one BS node, 10 master nodes, and 25 slave nodes. The BS connects to the computer through UART. The nodes with ID numbers from 1 to 10 are master nodes, and 11 to 35 are slave nodes. The slave node mounts a DS75 temperature sensor. It senses environment temperature and transmits the data every 5 minutes. Master nodes are not equipped with any sensors and are only responsible for data packet forwarding. We set the request beacon interval to be 0.5 s. The RF transmission power for slave node is −20 dBm, and for a master node it is −10 dBm. CENet drops a data packet after four retransmissions, which can be increased by the user to have a more reliable data yield.

The routing tree construction is completed within 30 seconds. The maximum number of hops of a routing tree is 4. Two criteria are adopted to estimate the link quality. The first one is the number of retransmissions, as described in Section 3.2.1, where the time window size is 12 data transmissions, *a* = 0.5, and *b* = 0.2. The second one is packet drop rate. The threshold of the packet drop rate is 10% in a time window.

**Figure 15.**
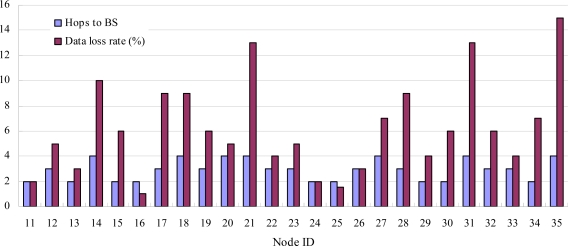
The average packet loss rate is 5.6% and nodes with more hops to BS have greater packet loss rate.

The network was running for 17 days. The BS received 115,056 data packets which are stored in a PostgreSQL [21] database. Because every data packet includes an attribute of sequence number, BS can calculate packet loss rate, as shown in Figure 15. The data packet loss rate varies between 0% and 15% with an average of 5.6%. From Figure 15, one can see that nodes with more hops have greater packet loss rate. This is because the retransmission mechanism in CENet only provides a certain reliability of transmission at each hop (hop-by-hop reliability). In other words, it can’t ensure end-to-end reliable packet delivery.

#### 5.2.2. Energy Consumption

**Figure 16.**
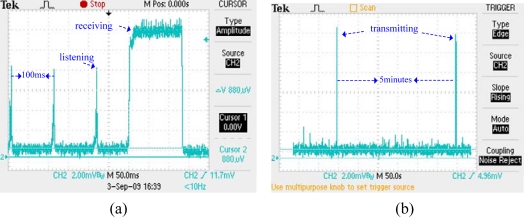
(a) Power consumption of master node. (b) Power consumption of slave node.

The energy consumption of master and slave node is measured by a Tektronix-2024B oscilloscope. Figure 16 illustrates the energy consumption of master and slave node. One can clearly see that master node listens to channel every 100 ms. On the other hand, the slave node sleeps most of the time and only wakes up for data packet transmission.

**Figure 17.**
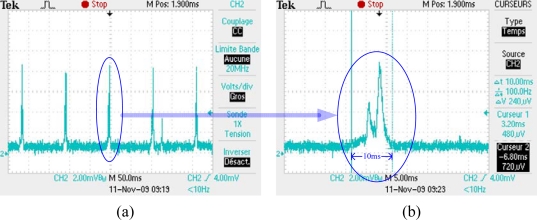
(a) Node listens to the channel for 10 ms with an interval of 100 ms. (b) The measure current in one listening period.

**Figure 18.**
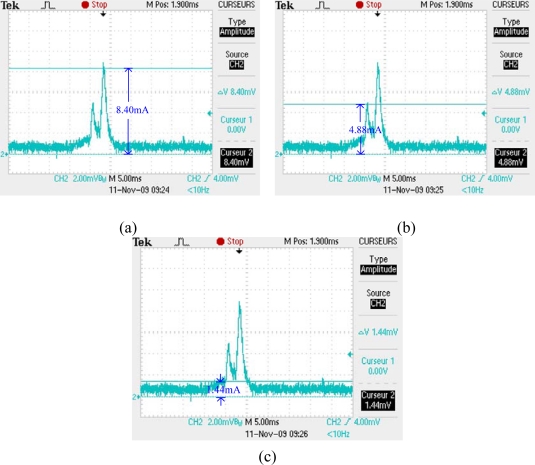
The detailed values of the measured listening current.

Using mode 4 of B-MAC, the average listening current of a slave node is measured. As shown in Figure 17, a node listens to the channel for 10 ms with an interval of 100 ms. Figure 18 shows the measured current values. The average listening current is calculated as 2.327 mA. Considering the environment noise, we set the average listening current to be 2 mA, which does not have any effect on our analysis.

#### 5.2.3. The Effect of Interference

To test how the interference affects data packet transmission reliability, we have done a series of experiments. A sensor network contains one BS, three master nodes and five slave nodes. The RF transmission power for each node is −20 dBm, and the sample interval for slave node is 30 s. Firstly, we placed the network in a sealed steel box. The external interference can be ignored. The link quality is good because they are close to each other [5]. The average delivery ratio of CENet and standard stack (standard TinyOS networking stack) are both 100%. Secondly, we placed the network in a laboratory and a three-hop tree is constructed. As shown in Table 5, the average delivery ratio of CENet is 94.5% and for standard stack it is 82.3%.

Finally, an interference node is introduced into the sensor network and its MAC protocol is modified by disabling the backoff mechanism. It continuously broadcasts data and does not consider the channel status (busy or idle). The interference is placed into the network. As shown in Table 5, CENet has delivery ratio of 89.2%. However, the delivery ratio of the standard stack is decreased to 64.7%. That is mainly because the MintRoute routing protocol cannot quickly repair an interfered route while CENet is robust to link interference.

## 6. Related Works

Many works have been done for environmental monitoring, for example, health monitoring of mechanical machines [22], volcano monitoring [23], earthquake monitoring [24], redwood monitoring [25], underground coal mine monitoring [26], structural health monitoring [27,28], and habitat monitoring [29]. However, current networks can’t satisfy the requirement of cabinet environmental monitoring which requires dense deployment and long network lifetimes.

Nasipuri *et al.* presented a wireless sensor network that is deployed in a substation for monitoring the health of power subsystems, such as circuit breakers, transformers, and transformer bushings [30]. They used a mixture of solar-powered nodes and battery-powered nodes to construct a hierarchical topology. However it is an outdoor deployment sensing network and can’t be adopted for cabinet environmental monitoring.

Genomote [2] is a project for data center monitoring, which is developed at Microsoft Research, where IEEE 802.15.4 wireless technology over wired networks and WiFi is adopted. Two classes of Genomotes are designed: master motes and slave sensors. A slave sensor connects with a master mote using a wired medium. Because of the partly wired connections, it is inconvenient for sensor deployment. In this project, a reliable data acquisition system [31] is developed and it can achieve 99% reliable data yield. Master motes are powered by computer through USB connection. Energy efficiency is not considered in the data acquisition system and thus it can’t be used in battery-powered wireless sensor networks.

## 7. Conclusions and Future Work

Cabinet environmental monitoring represents a typical application for wireless sensor networks. We have proposed CENet for cabinet environmental monitoring. CENet uses two kinds of nodes: slave and master, to construct a hierarchical architecture. The slave nodes are the front sensing tier and the master nodes are the communication infrastructure which is responsible for forwarding data from slaves to BS. CENet implements a data-aided routing protocol which achieves 93% delivery ratio with fewer beacon transmissions compared to periodic beaconing. In addition, using our proposed scheduling, a slave node stays in sleep mode in most of the time and only wakes up for sensing data transmission, which helps prolong network lifetime.

A CENet may consist of hundreds of nodes within high voltage cabinets in a substation. The communication interference can be serious. One way to increase data throughput and reduce data latency is using multiple BSs at different locations. Furthermore, we can take advantage of multi-channel sensor network to reduce interference. As future work, we intend to improve CENet to support multiple BSs and channel diversity.

## Figures and Tables

**Table 1.** Node parameters.

**Mote Type**	**Dot**	**Mica2**

Microcontroller	MSP430F1611	ATmega128
Radio	CC1101	CC1000
Data rate (kbps)	38.4	38.4
Power dissipation for reception (mA)	15.0	15.0
Power dissipation for transmission (0dBm) (mA)	16.0	16.0
Battery	A button battery	A pair of rechargeable batteries
Integrated sensors	Yes	No

**Table 2.** Example of estimator.

***i***	**1**	**2**	**3**	**4**	**5**
*C_i_*	8	7	10	7	9
*H_i_*	8	7.5	0	7	0
*T_i_*	9	9	0	8	0
Routing maintenance	No	No	Yes *C_3_* > *T_2_*	No	Yes *C_5_* > *T_4_*

**Table 3.** Energy consumption.

**Operation**	**Energy (*mJ*)**		**Time (s)**	

Receive a packet	5.74704	*E_rx_*	0.127	*t_rx_*
Transmit a packet	7.66272	*E_tx_*	0.127	*t_tx_*
Sense environment	66.0	*E_s_*	1.1	*t_s_*

**Table 4.** Related parameters.

**Notation**	**Parameter**	**Value**

*I_sleep_*	Sleep current (*mA*)	0.03
*I_listen_*	Average listening current (*mA*)	2.0
*V*	Voltage	3.0
*t_listen_*	Listening time (*s*)	0.01
*n*	Number of neighbors	3

**Table 5.** The average delivery ratio.

	**Sealed steel box**	**Laboratory**	**With interference**

CENet	100%	94.5%	89.2%
Standard	100%	82.3%	64.7%
